# Cell death pathways in pathogenic trypanosomatids: lessons of (over)kill

**DOI:** 10.1038/s41419-019-1370-2

**Published:** 2019-01-30

**Authors:** Rubem Figueiredo Sadok Menna-Barreto

**Affiliations:** Laboratory of Cell Biology, Oswaldo Cruz Institute, Oswaldo Cruz Foundation, Av. Brasil 4365, 21040-360 Manguinhos, Rio de Janeiro Brazil

## Abstract

Especially in tropical and developing countries, the clinically relevant protozoa *Trypanosoma cruzi* (Chagas disease), *Trypanosoma brucei* (sleeping sickness) and *Leishmania* species (leishmaniasis) stand out and infect millions of people worldwide leading to critical social-economic implications. Low-income populations are mainly affected by these three illnesses that are neglected by the pharmaceutical industry. Current anti-trypanosomatid drugs present variable efficacy with remarkable side effects that almost lead to treatment discontinuation, justifying a continuous search for alternative compounds that interfere with essential and specific parasite pathways. In this scenario, the triggering of trypanosomatid cell death machinery emerges as a promising approach, although the exact mechanisms involved in unicellular eukaryotes are still unclear as well as the controversial biological importance of programmed cell death (PCD). In this review, the mechanisms of autophagy, apoptosis-like cell death and necrosis found in pathogenic trypanosomatids are discussed, as well as their roles in successful infection. Based on the published genomic and proteomic maps, the panel of trypanosomatid cell death molecules was constructed under different experimental conditions. The lack of PCD molecular regulators and executioners in these parasites up to now has led to cell death being classified as an unregulated process or incidental necrosis, despite all morphological evidence published. In this context, the participation of metacaspases in PCD was also not described, and these proteases play a crucial role in proliferation and differentiation processes. On the other hand, autophagic phenotype has been described in trypanosomatids under a great variety of stress conditions (drugs, starvation, among others) suggesting that this process is involved in the turnover of damaged structures in the protozoa and is not a cell death pathway. Death mechanisms of pathogenic trypanosomatids may be involved in pathogenesis, and the identification of parasite-specific regulators could represent a rational and attractive alternative target for drug development for these neglected diseases.

## Facts

 

- The apoptotic phenotype occurs in trypanosomatids, but the precise molecular machinery involved and biological relevance must be further investigated.
- Autophagy was described in trypanosomatids, including Atg participation.
- Autophagy represents a parasite strategy for survival in stress situations, leading to cell death in extreme conditions.

## Open questions

 

- What is the real biological relevance of programmed cell death in protozoa?
- Which molecules participate in apoptotic-like activation/regulation in trypanosomatids?
- What are the molecular mechanisms involved in protozoan autophagy?
- Which molecules trigger/suppress autophagy in these protozoa?
- Are apoptotic-like and autophagic pathways good drug targets in trypanosomatids?

## Introduction

Neglected tropical diseases describe infective illnesses of poor populations, often in low-income countries, that affect one billion people worldwide^1^. Among these diseases, trypanosomatids-caused diseases are responsible for high annual mortality in tropical countries. These illnesses also show therapeutic complications, reinforcing the urgency of alternative medicines^2–4^. In the increased resistance scenario, improved comprehension of exclusive molecular mechanisms or biochemical pathways in these pathogens is an interesting strategy for future drug design. Here, different death processes of the pathogenic trypanosomatids were reviewed.

### *T. cruzi* and Chagas disease

Chagas disease is caused by the parasite *Trypanosoma cruzi*, and its transmission depends on contamination through triatominae vector faeces, blood transfusions, or even oral and congenital routes^5,6^. This disease affects eight million Latin America individuals;^7^ however, due to the immigration of infected people, together with contaminated food ingestion, Chagas disease has emerged in non-endemic countries^8,9^. This illness presents two clinical phases, including the acute phase characterized by high parasitaemia and a chronic phase that can be symptomatic or asymptomatic. Symptomatic chronic phase occurs in approximately 30% of infected people after extensive latency periods. The most remarkable clinical manifestations are observed in chronic patients, including dilated cardiomyopathy and/or digestive alterations^10^. Chemotherapy is based on two nitrocompounds that are very efficient in acute infections but show limited effectiveness in chronic phase, culminating in severe collateral effects. Chronic patients lose work productivity, representing an annual global economic burden^11^. The development of alternative drugs is mandatory and includes different classes of compounds and therapeutic strategies^12–15^.

*T. cruzi* presents a complex life cycle, including two hosts and different stages of evolution^16^. In triatomine midgut, epimastigote proliferates and adheres to the epithelium. After epimastigotes´ migration to the insect rectum, a differentiation is triggered by acid and low nutritional environmental conditions, and metacyclic trypomastigote (infective stage) is generated. After triatomine feeding, faeces containing metacyclics reach the mammalian bloodstream through wound openings or mucosa. Once in the vertebrate host, metacyclics can invade all nucleated cells, initiating a differentiation to amastigotes in the intracellular environment. Amastigotes replicate several times before differentiating into bloodstream trypomastigotes. This last stage ruptures the host cell, spreading the infection. The cycle closes when a non-infected triatomine bites an infected mammal^16^.

### *T. brucei* and sleeping sickness

Sleeping sickness is caused by *Trypanosoma brucei*, which has two clinically relevant subspecies: *T. b. rhodesiense*, which is related to acute zoonosis that occasionally infects man; and *T. b. gambiense*, which is responsible for chronic disease and 98% of all cases. Recently, the WHO estimated that 70 million people are at risk of infection and 30,000 new cases are emerging, despite initiatives to control this disease^17,18^. Sleeping sickness presents in two distinct phases. During the first phase, *T. brucei* is concentrated in the bloodstream and lymphatic system, and during the second stage, the protozoa cross the blood-brain barrier and reach the central nervous system, causing progressive neurological damage^19^. In the absence of adequate treatment, disease usually leads to death following clinical development in six months in the case of rhodesiense disease. Gambiense sleeping sickness, however, generally presents a chronic course up to three years in duration^20^. Early infections with *T. b. rhodesiense* and *T. b. gambiense* are usually treated with suramin and pentamidine, respectively^21^, while late infections depends on eflornithine or melarsoprol, drugs that have important limitations. Eflornithine is expensive and difficult to administer, whereas melarsoprol is extremely toxic and has demonstrated limited efficacy for *T. b. rhodesiense* infection^17^. In the last twenty years, efforts were made to develop a first-line treatment using a combination of melarsoprol and nifurtimox but the resistance especially to melarsoprol was a restriction^22^.

Non-replicative metacyclic forms initiate the life cycle when the tsetse fly *Glossina* spp bites the vertebrate, and *T. brucei* reaches the bloodstream. Differentiation occurs, and rapidly dividing slender forms are generated. Such forms evade the host immune system and avoid antibody binding through antigenic variation^23^. The cycle arrest induces slender forms to differentiate into short, stumpy parasites. During a tsetse blood meal, stumpy forms reach the fly’s midgut where the parasite differentiates to a proliferative procyclic form, that migrates to the insect salivary gland, resulting in new differentiation now to metacyclic form (infective stage), which closes the cycle^24^.

### *Leishmania sp.* and leishmaniasis

Leishmaniasis is a sand fly-borne disease caused by *Leishmania sp*, which infects 12 million people worldwide^25^. The clinical manifestations of leishmaniasis vary according to the individual host immune response and the infective parasite species^26^. Three clinical manifestations can be clearly observed: cutaneous (CL), muco-cutaneous (MCL) or visceral (VL, kala azar)^27^. In CL, an open, self-healing lesion occurs in the location of the sand fly bite. The most serious CL manifestation is the diffuse form where lepromatous lesions are disseminated throughout the skin and are difficult to heal. In MCL, mucosal membranes are affected, leading to facial disfiguration are also present in many cases. VL is the most dangerous clinical manifestation and is fatal if not treated. In this disease form, a strong inflammatory response occurs in the organs, especially the spleen and liver^28^. The compounds available for the clinical treatment are considered highly toxic, and increased drug resistance also represents a serious problem. For both CL and VL, the first choice drugs are the pentavalent antimonials, but due to the limited efficacy and severe side effects, resistance risk is increased considerably^29^. The second choice for VL is amphotericin B, which is also toxic and requires intravenous administration^30^. Today, miltefosine oral administration is restricted to VL treatment in India^31^, and the high cost, teratogenicity and side effects make this compound far from ideal^32^. Pentamine has been used in antimony-resistant VL cases as a second-line alternative, but its toxicity usually result in treatment abandonment^33^.

The life cycle of *Leishmania* involves two parasite forms and two hosts. In sand fly gut, non-infective procyclic and infective metacyclic promastigotes coexist^34^. After the insect blood meal, the metacyclic forms infect macrophages, differentiating into amastigotes responsible for parasite proliferation in the vertebrate^35^. Amastigotes are essentially intracellular and have adapted to survive in an acidic parasitophorous vacuole, whereas metacyclic promastigotes are resistant to the mammalian complement, which is a crucial characteristic to their survival during the initial infection.

## Cell death: an overview

Cell death is defined as the collapse of all metabolic processes triggered by chemical, physical or even natural stimuli and can lead to disease depending on the extent of damage^36,37^. In metazoans, cell death is an essential step for a great variety of physiological events, such as embryogenesis and tissue remodelling^38^. Almost a half-century ago, a non-accidental aspect of cell death was postulated, related to a sequence of orchestrated events without inflammation known as programmed cell death (PCD)^38^. More recently, different phenotypes associated with distinct PCD pathways have been proposed, including apoptosis, autophagy and necrosis, which is the most studied.

### Apoptosis

The term apoptosis (from the Greek “falling off”) was created in the early 1970s to classify a crucial PCD process that occurs during metazoan embryo growth^39^. Actually, this pathway is essential for many biological processes, including the removal of non-functional or damaged cells in all tissues^40^. This early and efficient removal prevents the inflammatory response^41^. In a pathological scenario, apoptosis plays a fundamental role in cellular defence, composing a mechanism to control pathogen dissemination and cancer development^42–45^.

In metazoans, the apoptotic machinery is triggered by intrinsic or extrinsic factors^36^, resulting in activation of cysteine-dependent aspartate-directed proteases known as caspases that lead to the apoptotic phenotype^46^. The extrinsic pathway starts with the binding of death ligands (FasL, TNF-α, among others), which are soluble and/or present on the effector cells surface, to their respective receptors located in target cells, which triggers the caspase 8 activation and subsequent procaspase 3 cleavage. Following the cascade process, caspase 3 activates endonuclease G, leading to an internucleosomal DNA fragmentation, which is one of the most important hallmarks of apoptosis^47,48^. Two different mechanisms are related to the intrinsic pathway and involve participation of mitochondria or even the endoplasmic reticulum (ER). Activation of mitochondrial pathway derives from the formation of a pore in the outer membrane of this organelle that allows cytochrome c release, as well as the release of other mitochondrial molecules such as endonuclease G, apoptosis induction factor and Bcl2 proteins to the cytoplasm. The interaction among cytochrome c, procaspase 9 and apoptotic protease activating factor 1 forms the apoptosome in the cytoplasm, which promotes the procaspase 9 cleavage, and caspase 9 activates caspase 3^49,50^. The ER pathway depends on caspase 12, but controversial data about the functional role of this pathway in humans up to now make the biological importance of this pathway debatable^51,52^.

Apoptotic regulation is very complex and includes many anti- and pro-apoptotic molecules that negatively or positively control the pathway. Many of these regulators are members of the Bcl-2 family, such as Bcl-2 and Bcl-xL anti-apoptotic proteins. Other crucial molecular checkpoints are also present in mammalian cells^53^. Proteins such as the endogenous inhibitor of apoptosis (IAP) and the inhibitor of IAP known as smac/DIABLO can change cell fate after triggering cell death signalling^54,55^. As an example of other apoptotic inhibitors, the overexpression of prohibitin, a protein ubiquitously expressed in mitochondria, partially blocks the mitochondrial apoptotic pathway^56^, and the inhibition of inosine 5′-monophosphate dehydrogenase (purine metabolism enzyme) also induces apoptosis^57^. The precise balance of anti- and pro-apoptotic molecules and activation is crucial for apoptotic success^58^.

Among the apoptotic phenotypes, caspase activation, typical internucleosomal DNA fragmentation, blebs in the plasma membrane (apoptotic bodies), cell shrinkage, mitochondrial membrane potential (ΔΨm) loss and phosphatidylserine (PS) exposure are the most relevant^59^. Due to the pivotal role of caspases, the gold-standard method is analysis of the specific cleavage of these protease substrates. The evaluation of caspase activity by immunoassays (ELISA, flow cytometry, western blotting) or even the use of the inhibitors and/or specific labelled substrates are also good alternatives. DNA fragmentation can be investigated using terminal dUTP nick-end labeling (TUNEL) technique or electrophoresis (laddering profile in gel). PS externalization (annexin V/ propidium iodide assay) and ΔΨm loss (rhodamine 123, tetramethylrhodamine ethyl ester, tetramethylrhodamine methyl ester or derivatives labelling) are also well-employed^60,61^.

### Autophagy

More than fifty years ago, Dr De Duve’s group suggested the presence of a physiological process for self-digestion of non-functional organelles and/or macromolecules named autophagy (from Greek: *auto*—self and *phagein*—to eat)^62^. Autophagy represents a pathway involved in turnover and recycling by removal of damaged cellular components, regulating homeostasis during crucial processes such as cell growth and differentiation^63^. Autophagy is usually exacerbated under pathological conditions and/or pathogenic infections^64,65^. On the other hand, continuous autophagic induction can lead to a breakdown of the cellular balance, inducing autophagic cell death^66^.

The first autophagy-related genes (ATGs) were described in *Saccharomyces cerevisiae*. Nearly thirty proteins were identified, and their participation in different points of the pathway was proposed^67^. Today, the autophagic molecular machinery has been demonstrated to be highly conserved and Atg orthologues are distributed among all eukaryotes, and Atg8 (LC3 in mammals) detection is considered the gold-standard method for monitoring autophagic flux^68^. Macroautophagy, microautophagy and chaperone-mediated autophagy (CMA) are the three types of autophagy currently described. In macroautophagy (also called autophagy), the intracellular material that will be degraded is surrounded by a double membrane structure known as the phagophore, which forms the autophagosome that directs the cargo for lysosomal degradation^69,70^. The autophagic initiation is dependent on the serine/threonine protein kinase TOR (target of rapamycin) that functions as a nutrient sensor and on a phosphatidylinositol 3-kinase (PI-3K) known as Atg6 (beclin 1 in mammals)^62^. In microautophagy, the cargo is engulfed by an invagination of the lysosomal membrane, which forms small single-membrane vesicles inside lysosomes, demonstrating an ultrastructural aspect of multivesicular bodies. The absence of specific markers for microautophagy makes this autophagic type poorly described. In CMA, target proteins bind to cytoplasmic chaperones by pentapeptide motifs (KFERQ, VDKFQ or QREFK), making this autophagic type the most selective. The binding of chaperone-substrate to the lysosomal receptor LAMP-2A leads to the receptor dimerization and subsequent channel formation. By this channel, the target molecule enters into the organelle to be degraded^62,71^.

Since 1964 when the term autophagy was coined, up to now, ultrastructural analysis remains an important tool for the characterization of autophagic phenotypes. As mentioned above, the detection of Atg8/LC3 in microscopy approach (LC3 puncta) and/or by western blotting (LC3-I and LC3-II detection) is the most widespread method to confirm autophagic activity. In parallel, the use of pharmacological inductors or inhibitors (PI-3K inhibitors and/or rapamycin) as well as Atg or related protein knock down or knock out models are also extensively employed^68^.

### Necrosis

Indeed, cells can also die accidentally as in the case of extensive injury due to external stresses such as mechanical disturbance, drugs or infection, and other factors. These stimuli induce random cellular degradation, culminating in the rupture of the plasma membrane, which leads to an intense inflammatory response. Necrosis (in Greek, “dying stage”) is defined as accidental cell death^72^. The necrotic phenotype involves plasma membrane and calcium homeostasis disruption, as well as lysosomal hydrolase-dependent degradation and cytoplasmic vacuolization. The loss of cellular integrity promotes the release of damaged organelles and/or intracellular molecules and induces inflammation, which is one of the most remarkable differences between necrosis and apoptosis^73^.

## Death processes in trypanosomatids

The role of PCD in multicellular organisms has been extensively described in the last fifty years and is associated with homeostasis regulation. However, despite higher and lower eukaryotes sharing related biochemical mechanisms and molecular events, remarkable differences are clearly observed and should be carefully evaluated^74,75^. Actually, the biological relevance of PCD for protozoa has not yet been demonstrated. In the following sections, death events in these parasites associated with apoptosis-like cell death, autophagy and/or necrosis as well as their induction by extrinsic or intrinsic factors will be reviewed with a focus on the importance of PCD for the biology of pathogenic trypanosomatids.

### Apoptosis-like cell death in trypanosomatids

**Fig. 1:**
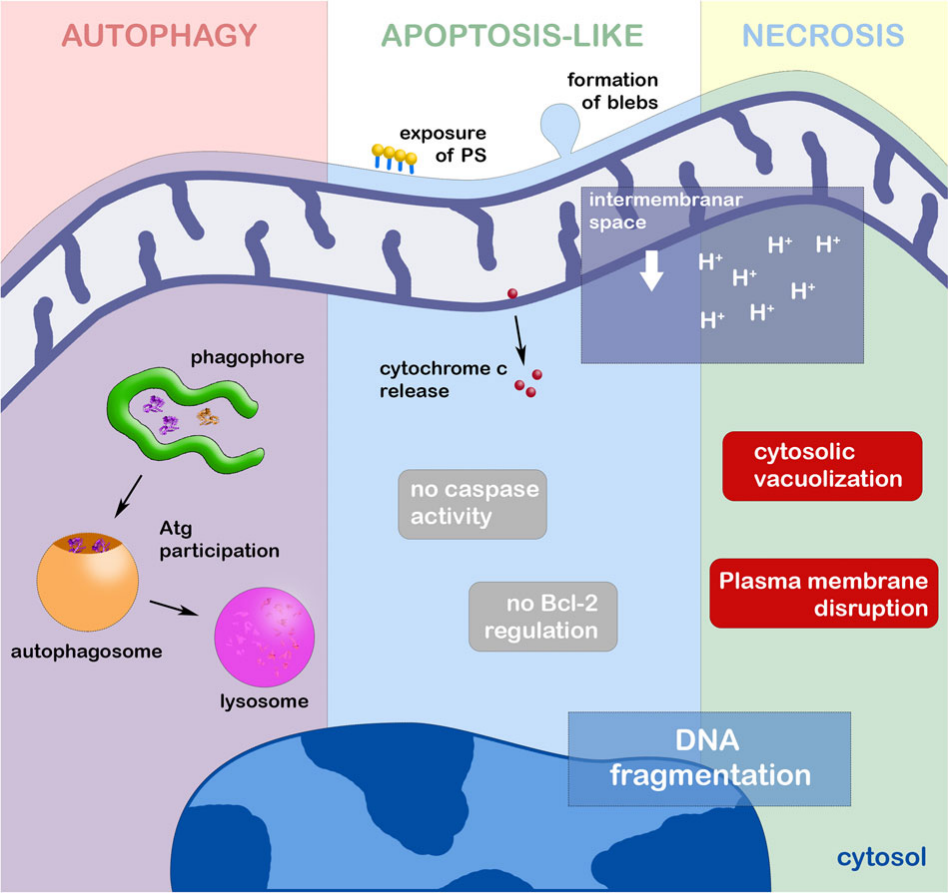
Schematic representation of three most important cell death processes occurring in pathogenic trypanosomatids. After the autophagic induction, phagophore elongates from endoplasmic reticulum or Golgi profiles, engulfing non-functional organelles and molecules. After the elongation step, autophagosome is formed, taking the cargo to be degraded in lysosome. All steps are regulated by Atg proteins. Autophagic cell death occurs when the homeostatic balance is broken by the continuous autophagic induction. In trypanosomatids, apoptosis-like cell death is characterized especially by the effect on the mitochondrion, with loss of its membrane potential, and release of cytochrome c to the cytosol. Among other classical apoptotic phenotypes, DNA fragmentation (derived from EndoG activity) and PS exposure were also described in these protozoa. The participation of metacaspases is still debatable as well as the presence of apoptotic Bcl-2 family regulators. Necrotic pathway is evidenced by the intense cytosolic vacuolization, strong effect on the mitochondrion, randomic DNA fragmentation and plasma membrane disruption

Only three decades after it was first described, PCD was suggested in trypanosomatids. Based on the mammalian apoptotic phenotype, alterations in nuclear morphology and DNA fragmentation were observed during *T. cruzi* differentiation^76^. After this first description, many authors have reported apoptotic features induced by stress conditions such as drug treatment, heat shock, and others^76–82^. Moreover, in pathogenic trypanosomatids, the identification of an apoptosis-like phenotype is restricted to DNA fragmentation, PS externalization, ΔΨm loss and cytochrome c release, which are typical markers of apoptosis in higher eukaryotes (Fig. 1)^76,78,83,84^. However, pivotal molecules involved in PCD regulation have not yet been described^85^. These gene sequences could differ from the classical mammalian genes, and the inability to find homologues could represent a problem for the molecular proposal in trypanosomatids.

**Fig. 2:**
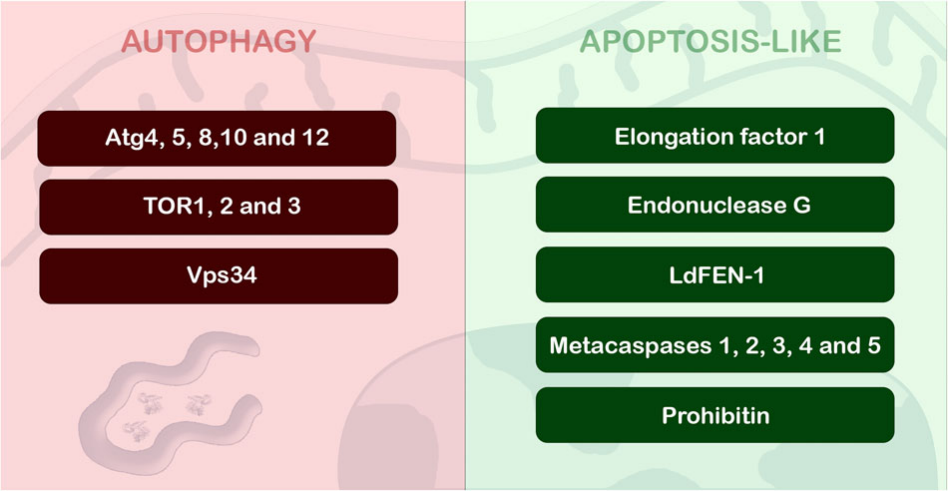
Autophagic and apoptotic molecules detected in pathogenic trypanosomatids. Five Atg proteins, Vps34 and also three TOR were described and associated with autophagic process in these parasites. In relation to apoptosis-like, endonuclease G, prohibitin, elongation factor 1 and LdFEN-1 have been associated to this death machinery. The importance of metacaspases for apoptosis-like of trypanosomatids is too controversial, and their molecular function is still under investigation

**Fig. 3:**
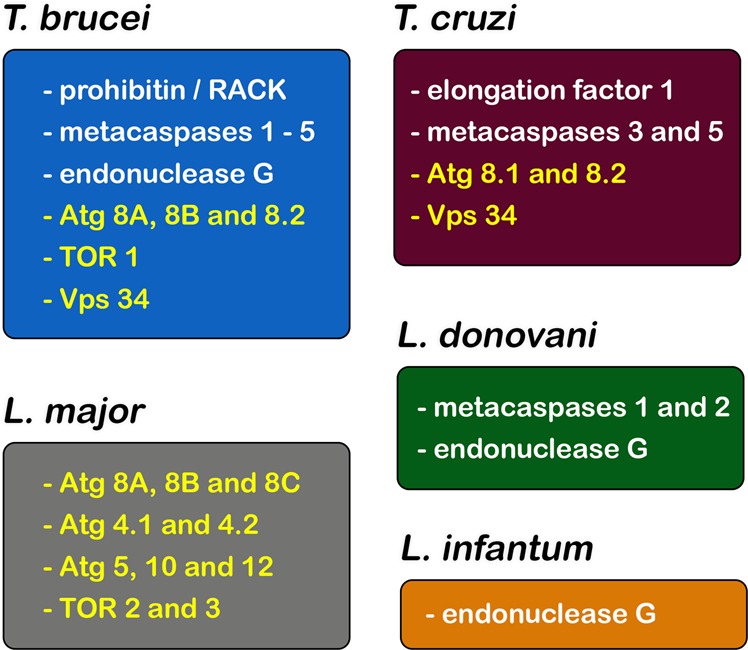
Distribution of cell death molecules in different species of pathogenic trypanosomatids. Proteins associated with apoptosis-like (white) and autophagy (yellow). Apoptosis-related molecules such as endonuclease G and metacaspases were mainly studied in *T. brucei* and *T. cruzi*, followed by *L. donovani* and *L. infantum*. On the other hand, autophagic molecules were extensively assessed in *L. major*, and also in *T. brucei* and *T. cruzi*

In *T. brucei*, an apoptotic phenotype was described in parasites incubated with cytokines, drugs and even ROS^86–88^. The correlation between activated protein kinase C receptor, prohibitin and apoptosis-like cell death was proposed, indicative of convergence between the mammalian and parasite pathways (Figs 2 and 3)^86^. Despite metacaspases and mammalian caspases sharing similar folding patterns, the presence of evidence regarding caspase-like activity on their respective substrates has not been detected in pathogenic trypanosomatids^83,89,90^. In this context, the detection of the self-proteolytic activity of *L. major* metacaspase was demonstrated in vitro (Figs 2 and 3)^91^. Cleavage of crucial substrates by metacaspases and its involvement in PCD has not been reported thus far^92,93^. Furthermore, the participation of metacaspases has been associated with parasite proliferation and differentiation^93–95^.

The classical apoptotic features described above could be detected in *Leishmania* sp. under non-physiological conditions such as drug treatment, nutritional deprivation or oxidative stress^74,83,88,90,96,97^. DNA fragmentation was detected in *L. major*, *L. mexicana* and *L. donovani* promastigotes and axenic amastigotes in total DNA electrophoresis, but the ladder pattern suggestive of internucleosomal cleavage was unclear. The presence of Mg^2+^- and Ca^2+^-independent endonucleases also helps to support the hypothesis of a classical DNA fragmentation pattern^78,89,98^. Furthermore, a metacaspase-independent death pathway has been described and involves endonuclease G-like and mitochondria (Figs 2 and 3)^96,98,99^. The involvement of inosine 5′ monophosphate dehydrogenase in an apoptosis-like cell death phenotype was also suggested in *L. amazonensis* after heat shock based on PS exposure and mitochondrial depolarization (Fig. 1), but further analysis regarding the molecular mechanism must be performed before any conclusions are made^100^.

ROS generation and mitochondria also play an essential role in the PCD phenotype in unicellular eukaryotes^101^. Hydrogen peroxide leads to ΔΨm loss and DNA fragmentation in *L. donovani*. Partial reversion of the phenotype by classical caspase inhibitors and the detection of caspase-like activity also reinforced these findings^83,96^, but careful analysis of the proteolytic activity of metacaspases must be conducted to confirm their role in the cell death pathway. The imbalance of Ca^2+^ influx in the mitochondrion triggers oxidative stress in trypanosomatids^102^, and prostaglandin D2 induces ROS production in *T. brucei*, which leads to an apoptotic-like cell death phenotype that is partially reverted by antioxidants^84,103^. In the following sections, oxidative involvement in autophagy and necrosis will also be debated^75,104^. On the other hand, transfection with the mammalian Bcl-XL gene partially reverted apoptosis-like cell death in *L. infantum* after heat shock despite a lack of detection of caspase-like activity^105^. Up to now, Bcl-2 family members have not been identified in pathogenic trypanosomatids (Fig. 1)^86^. Further investigation regarding apoptosis-like cell death regulatory steps must be performed. Comparisons between mammalian and protozoan death phenotypes are summarized in Table 1.

**Table 1:** Comparison between death phenotypes in metazoans and trypanosomatids

Phenotypes	Metazoans	Trypanosomatids
*Apoptosis*		
no inflammatory response	+	na^a^
proteolytic activity of caspases	+	nd^b^
Δψm dissipation	+	+
cytochrome c release	+	+
Bcl-2 proteins regulation	+	nd
blebs formation in plasma membrane	+	+
internucleosomal DNA fragmentation	+	na
PS exposure	+	+
cell shrinkage	+	+
*Autophagy*		
autophagosomes formation	+	+
Atgs regulation	+	+
TOR and PI-3K participation	+	+
degradation in lysosomes	+	+
presence of KFERQ, QREFK or VDKFQ motifs in the target protein (only in CMA)	+	nd
*Necrosis*		
inflammatory response	+	na
plasma membrane rupture	+	+
cytosolic vacuolization	+	+
calcium misbalance	+	+
lysosomal enzymes release	+	+

^a^na: not applicable

^b^nd: not determined

### Autophagy in trypanosomatids

In trypanosomatids, the first ultrastructural description of an autophagic process occurred more than four decades ago in *T. brucei*^106^. Stress conditions, especially drug treatment, frequently induced an autophagic phenotype such as myelin-like structures, multivesicular bodies and an increase in autophagosome number^82,107–111^. The most recurrent autophagic evidence found in stressed parasites was myelin-like structures and concentric membrane structures morphologically similar to phagophores, the isolated membrane that originates autophagosomes (Fig. 1). ER profiles are usually reported as the main membrane resource for phagophores, including in trypanosomatids. In *T. cruzi* epimastigotes, the participation of reservosomes (lysosome-related organelles) in autophagy has been proposed, and close contact between these organelles and ER profiles was also observed in the autophagic phenotype^84,110^.

The autophagic molecular machinery, including ATG homologues, is partially present in trypanosomatids, but many components of the yeast pathway are lacking in protozoa. Bioinformatic analysis showed genes that participate in phagophore expansion to degrade the autophagosome content, including the complete Atg8 conjugation system (Atg3, Atg4, Atg7 and Atg8)^112^. In 2008, Alvarez and co-workers demonstrated for the first time the biological role of Atg8 in *T. cruzi*, and autophagy was associated with a crucial step in parasite differentiation, which was essential for the success of this protozoa life cycle. This study pointed to the presence of two Atg8 (TcAtg8.1 and TcAtg8.2) and two Atg4 (Atg4.1 and Atg4.2) homologues. Interestingly, both Atg4 homologues, but only Atg8.1, recovered autophagy in knockout yeast strains. When the parasite was submitted to starvation, Atg8.1 was located in autophagosomes, validating this protein as an autophagosomal marker, as had already been done in mammals and yeast^70^. Four isoforms of Atg8 have been described in other pathogenic trypanosomatids (Atg8, Atg8A, Atg8B and Atg8C), and the dynamics of these molecules are also controlled by the same two Atg4 isoforms^113–115^. Moreover, the Atg12 conjugation system is defective in these parasites, and different genes are absent, such as ATG5, ATG10 and ATG12 (Figs 2 and 3)^112,116^. TOR kinases (TOR1 and TOR2) and their respective complexes (TORC1 and TORC2) were present in the genomes of pathogenic trypanosomatids, but their biological functions are still unclear due to the differences in molecular behaviour, subcellular localization and susceptibility to rapamycin^117^. Rapamycin induced the formation of a huge number of autophagosomes by TORC2 inhibition in *T. brucei*, thereby impairing parasite proliferation, but did not inhibit TORC1^117,118^. Additionally, in starved *T. cruzi*, TcVps15 overexpression increased the number of autophagosomes, suggesting a regulatory role for this enzyme in the control of TcVps34 enzymatic activity (Figs 2 and 3)^119^. Curiously, a great number of large-scale proteomic studies in trypanosomatids did not find evidence of autophagic proteins under nutritional stress (during metacyclogenesis, for example), strongly suggesting the inability to find homologues of Atgs in these parasites^120^.

The fundamental role of autophagy in the differentiation processes of trypanosomatids has been proposed, as well as in phospholipid homeostasis and mitochondrial functionality^70,121–123^. In 2012, Williams and colleagues produced *L. major* promastigotes lacking Atg5, and such mutants did not form autophagosomes but were still viable. Curiously, a strong reduction in virulence of these parasites was observed in vitro and in vivo, together with peculiar morphological changes, especially in flagellar length. In these mutants, a remarkable increase in phosphatidylethanolamine levels was detected as well as mitochondrial swelling with a decrease in this organelle membrane potential and high ROS content, suggestive of mitochondrial dysfunction derived from conjugation of mitochondrial PE to ATG8 for autophagosome biogenesis^122^. During metacyclogenesis, parasites are submitted to nutritional stress, a classical model of autophagic induction. The participation of this pathway in the control of virulence and infectivity to the mammalian host was demonstrated^121^. Especially in the triatominae rectum, nutritional deprivation represents a fundamental step for *T. cruzi* differentiation and has been previously shown to reduce Atg8.1 levels during metacyclogenesis^70,84^. However, further elucidation of the molecular machinery involved must be performed.

Selective autophagy was also reported in pathogenic trypanosomatids. *T. brucei* bloodstream forms differentiate into procyclic forms, and the selective degradation of glycosomes was postulated to occur during this process. Due to the biological importance of this peroxisome-like organelle for the glycolytic pathway, its degradation directly affects parasite bioenergetics, reinforcing the relevance of pexophagy in these protozoa^124,125^. It is well-known that environmental conditions (temperature, pH, among others) as well as nutritional availability (sources of energetic substrates) are different in distinct hosts, leading to changes in the parasite metabolic pathways, including ATP production^116^. Some of these metabolic adaptations could be regulated by autophagy. For example, the acidification of acidocalcisomes (organelles involved in polyphosphate metabolism) was directly related to autophagic regulation. In *T. brucei*, autophagy was blocked by the impairment of acidocalcisome biogenesis, suggesting that this organelle participates in this pathway^126,127^.

As described for trypanosomes, metacyclogenesis of *Leishmania* sp. is also dependent on the presence of autophagosomes^128,129^. The impairment of autophagic flux by Atg4.2 deletion and subsequent lipidated Atg8 accumulation has been associated with the reduction of promastigotes during differentiation^119^. Likewise, the involvement of autophagy was also reported during *L. mexicana* differentiation of metacyclic promastigotes into amastigotes^128^. The participation of lysosome-like organelles known as megasomes in the differentiation of this parasite has been described, as well as the proteolytic activity of two megasomal cysteine peptidases (CPA and CPB) related to the autophagic process. Deletion of these proteases deeply compromises the differentiation to amastigotes, leading to a notable increase in autophagosome number^116,128^. Table 1 also describes autophagic phenotypes observed in mammals and pathogenic trypanosomatids.

The term “autophagic cell death” is usually employed for situations where homeostatic control is lost and autophagy is exacerbated to degrade damaged structures, macromolecules or organelles^37^. One of the most usual experimental protocols to analyse autophagic cell death is preincubation with PI-3K inhibitors (wortmannin and 3-methyladenine), which are used to block the initial steps of this pathway, before the autophagic stimulus is initiated. Using this experimental design, our group showed that *T. cruzi* autophagy is part of the mechanism of action of naphthoimidazoles and involves the Atg8 conjugation system. However, the molecular mechanisms of death deserve more investigation^62,110^.

### Necrosis in trypanosomatids

Similar to the process that occurs in mammalian cells, necrotic cell death is poorly investigated in trypanosomatids. The main reason is the uncontrolled and accidental profile of this process, as it is always the endpoint of all death processes (PCD or not). It is well-established that plasma membrane disruption is the most characteristic necrotic event (Fig. 1) and is triggered by chemical and/or physical stress^73^. In vitro, in the absence of host phagocytosis, the parasites will rupture necessarily, no matter what stimulus or death process is induced. Preclinical tests have shown parasite lysis as the endpoint in the mode of action of a great variety of anti-trypanosomatid drugs^28,85,130–133^.

Activation of the complement cascade usually induces necrosis in trypanosomatids. It was previously reported that the binding of lectins to surface molecules of the parasite, such as glycosylated proteins or lipophosphoglycans, in the case of *T. cruzi* metacyclic trypomastigotes and *Leishmania* sp. promastigotes, respectively, triggered necrosis^134,135^. Furthermore, different evasion mechanisms are present in trypanosomatids to avoid the complement system. For example, the huge repertoire of variant surface glycoproteins provides a coat for *T. brucei*, allowing it to evade host immune defences^136^.

Another relevant issue concerning necrosis in these protozoa is related to oxidative stress. One of the regulators of ROS-dependent cell death is the mitochondrial permeability transition pore. In *T. cruzi*, the participation of mitochondrial cyclophilin as a component of this permeability pore was previously demonstrated. Incubation with hydrogen peroxide led to a loss of mitochondrial membrane potential and probably subsequent extensive lipid peroxidation in the parasite^137^. Depending on the ROS levels produced, protozoa lysis and consequent necrosis will occur.

## Conclusions

Even though PCD has been the subject of a great number of studies in trypanosomatids, the precise biochemical and molecular processes involved as well as the regulatory steps required are still unknown. The lack of key molecules in these parasites points to the existence of PCD as controversial and makes the term “apoptosis-like” more opportune^84,138^. Up to now, no evidence showing proteolytic activity of metacaspases in PCD events has been presented. Actually, all deep studies about orthologues of these proteases in trypanosomatids presented no correlation with death mechanisms, and their activity has been associated with proliferation and differentiation^85,94,139^. Indeed, remarkable differences between higher eukaryotes and trypanosomatids were detected, including a lack of key genes that encode molecular executioners and/or regulators in these protozoa^85^. Another crucial point was the inability to find homologues of cell death genes probably due to the low sequence similarities to metazoans. Previous data showed a conserved aspect of PCD, indicating its pivotal role in these species’ survival during evolution. Notwithstanding, in lower eukaryotes such as trypanosomatids, the identified death molecular mechanisms have been related to a divergent evolutionary event based on a huge phylogenetic analysis of death molecules^140^. Comparing protozoa and metazoans, important differences (including the absence of PCD molecules) were found in these parasites^141^. Despite all morphological evidence, up to the convincing identification of the executioners, Proto and co-workers (2013) classify this protozoan cell death as an unregulated process or incidental necrosis^85^.

Furthermore, the physiological relevance of this cell death type for pathogenic trypanosomatids is still an unanswered question. Altruism has been proposed as a hypothesis to explain PCD in unicellular eukaryotes^84^. Crucial events such as clonal selection, population density control or even evasion of host immune defences were associated with PCD, directly supporting the parasite life cycle^62,78,89,142^. It was suggested that trypanosomatid colonization is regulated by nutritional availability and protozoa cell death in the insect gut, controlling the parasite super-population and invertebrate host death^74,84^. Invertebrate forms of *L. amazonensis* or *T. cruzi* could suffer PCD to facilitate the infection by avoiding parasitic necrosis and immune response exacerbation^76,78^. As the altruistic hypothesis is difficult to prove, the uncontrolled status of cell death should be considered as the only possibility.

A critical autophagic role in cell death has also been proposed, but the regulatory steps involved are unknown^107,110,143^. In unicellular eukaryotes, few studies about autophagy-related events have been performed, and the molecular pathways are still unclear. Indeed, the autophagic process contributes to the maintenance of trypanosomatid homeostasis, despite the description of its participation in dying parasites. The imbalance in autophagic turnover of essential cellular factors could trigger death signalling; however, activation of this machinery could result from general and unspecific damage^143,144^. The recycling of non-functional and/or injured cellular components represents a central step in the direction of trypanosomatid survival, explaining the appearance of autophagic phenotypes induced by distinct drugs, as a parasite attempts to survive in stress conditions by degrading damaged structures^109,143,145,146^. On the other hand, the existence of a novel and alternative PCD pathway as well as cross-talk between more than one cell death process cannot be discarded in these protozoa, but it must be proved molecularly^58,66,75,109,110^.

Unfortunately, no biochemical or molecular tools for protozoa PCD characterization are commercially available. The absence of specific antibodies or enzymatic kits for the identification of apoptotic-like or autophagic events delays the development of more studies in this area. Furthermore, the death mechanisms of pathogenic trypanosomatids may have implications in their pathogenesis and deserve more investigation in the near future.
